# Unveiling an oral hemangiolymphangioma

**DOI:** 10.4322/acr.2023.435

**Published:** 2023-05-24

**Authors:** Rívea Inês Ferreira-Santos, Kamilla Alves Santos, Alexandre Prado Scherma, Jorge Esquiche León, Estela Kaminagakura

**Affiliations:** 1 Universidade Paulista (UNIP), Department of Oral Radiology, Campinas, SP, Brasil; 2 Universidade Estadual Paulista (UNESP), Institute of Science and Technology, Department of Biosciences and Oral Diagnosis, São José dos Campos, SP, Brasil; 3 Universidade de Taubaté (UNITAU), Department of Dentistry, Taubaté, SP, Brasil; 4 Universidade de São Paulo (USP), Dental School, Department of Stomatology, Public Oral Health, and Forensic Dentistry, Ribeirão Preto, SP, Brasil

**Keywords:** Angiokeratoma, lymphangioma, COVID-19, tongue, case reports

## Abstract

Hemangiolymphangioma is a very rare vascular malformation that develops as a combination of dilated venous and lymphatic vessels. We describe an unusual case of hemangiolymphangioma of the tongue affecting an adult man who complained of an uncomfortable, slowly progressing exophytic irregular dark red-violaceous nodular mass on his tongue that impaired speech and swallowing for two weeks. The clinical differential diagnoses were Kaposi’s sarcoma and a COVID-19-related lesion. A complete blood count and serology for HIV-1 and 2 and RT-PCR for COVID-19 were requested and results were negative. An incisional biopsy was performed. Microscopically, the lesion exhibited several dilated vessels lined by normal-appearing endothelial cells, some filled with prominent intravascular erythrocytes and others containing proteinaceous eosinophilic material resembling lymphatic vessels, in close association with hyperkeratosis, papillomatosis, and acanthosis. From immunohistochemical analysis, most vessels were found to be CD34 positive, some highlighted by α-SMA, whereas D2-40 was focal. Positive staining for some lymphatic and blood vessel markers, *i.e.*, D2-40 and CD34, respectively, indicates a mixed derivation of the lesion. HHV-8 was negative. Clinical features, the congested blood vessels with ectasia in intimate association with hyperplastic epithelium, and the immunohistochemical profile supported the final diagnosis of oral hemangiolymphangioma. The patient underwent minimally invasive surgical excision with no intercurrences. After 18 months of follow-up, there were no signs of relapse.

## INTRODUCTION

Hemangiolymphangioma is a very rare vascular malformation.^1-3^ In vascular tumors, blood vessel architecture is incomplete and surrounded by hyperplastic cells.^4,5^ On the other hand, vascular malformations consist of progressively enlarging ectatic vessels composed of veins, lymphatic vessels, venules, capillaries, arteries, or mixed vessel types.^4,5^ Histologically, different combinations of vascular elements, such as lymphatic and venous endothelium, may be seen in the same lesion,^3,4^ and these vascular spaces may be filled with red blood cells and proteinaceous fluid similar to lymph fluid.^3,5^ These mixed vascular malformations are termed ‘hemangiolymphangioma’ or ‘lymphangiohemangioma’ according to the prevalent vessel structure.^3^

Clinically, hemangiolymphangiomas may be diagnosed as red-to-violaceous plaques, papules, or nodules.^3,4,6-8^ Tongue lesions may increase in size resulting in macroglossia, which can lead to breathing, mastication, deglutition, and speech dysfunction.^6,8,9^ Oral lesions may resemble hemangioma,^3,7,10^ lymphangioma,^3,7,10,11^ angiokeratoma,^10^ pyogenic granulomas,^3,7,11^ Kaposi’s sarcoma,^12^ and purple vesiculobullous lesions and nodules as in COVID-19 patients.^13^

Microscopically, hemangiolymphangiomas, like other vascular malformations, do not show active cellular proliferation,^3^ but appear as subepithelial enlarged channels (veins, capillaries, arteries, and lymphatic vessels).^2,3,7^ Just beneath the stratified squamous epithelium with elongation of rete ridges, there are multiple dilated vessels lined by normal-appearing endothelial cells.^1,5^ Vessels are capable of accumulating fluids. In hemangiolymphangiomas, most vascular channels contain erythrocytes, with or without thrombi formation, partly or completely enclosed by papillomatous epithelial tissue.^1,6^ Inflammatory components are not observed in the specimens.^5,11^ The definite diagnosis of hemangiolymphangioma may require investigation of clinical, microscopic, and immunohistochemical features.^6,10,14,15^ In addition, clinicians must search for familial medical history and assess the results of laboratory tests to exclude genetic alterations, HIV infection, as well as COVID-19 with its systemic and oral involvement.^3,6,7,9,12,13^ Remarkably, the tongue appears to be a frequent site for COVID-19-related oral manifestations.^13^

In contrast to hemangiomas, spontaneous regression of hemangiolymphangiomas is rarely observed.^5,14^ Various therapeutic approaches have been proposed based on the size, type, and location of a lesion, as well as its association with anatomic structures and infiltration to the surrounding tissues.^4,5,7,8,11,14,16^ Nevertheless, complete surgical excision is still the usual treatment option for these lesions whenever possible.^5,11^

This clinical case report comprises a survey of published cases and adds information to the scant literature on oral hemangiolymphangiomas.

## CASE REPORT

**Figure 1:**
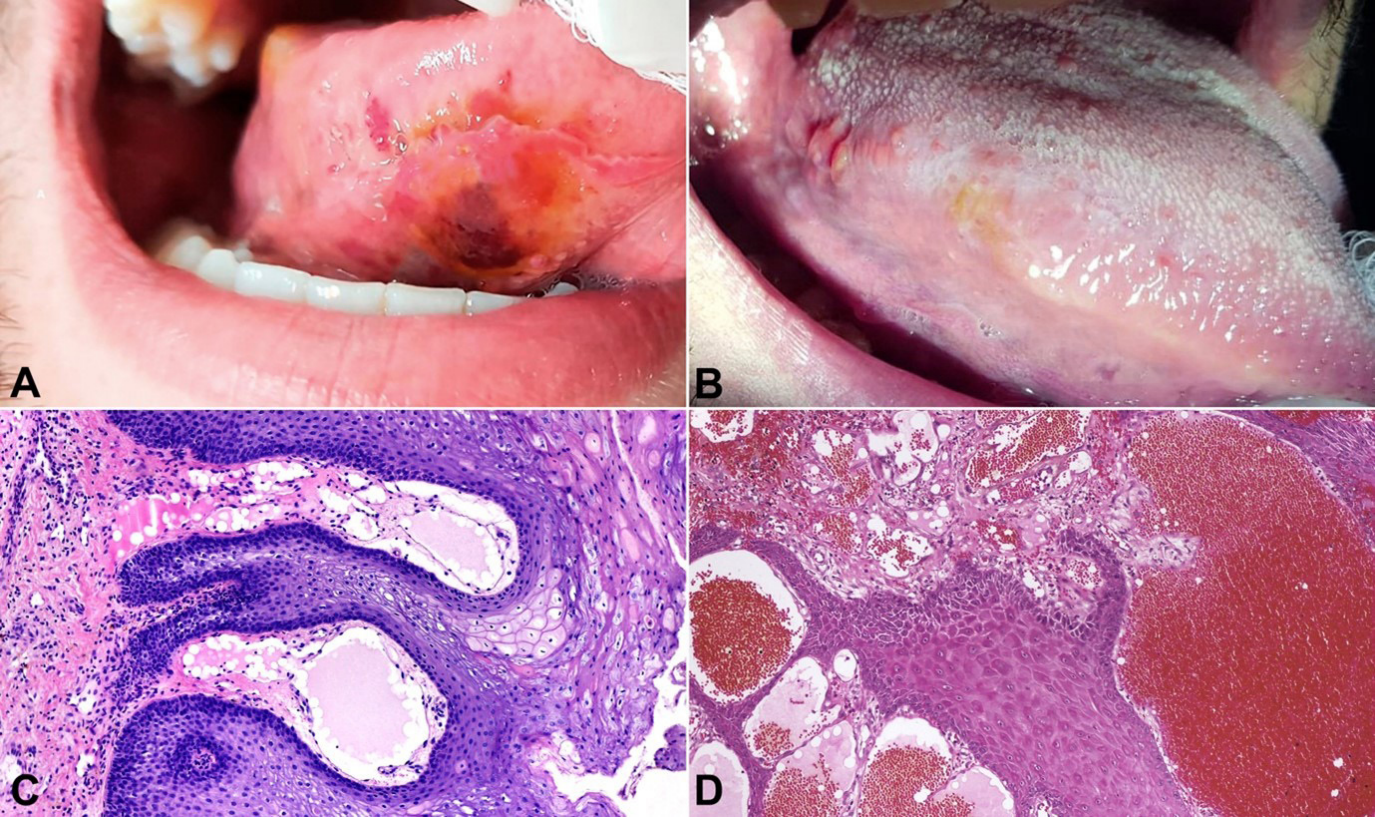
Preoperative *vs* postoperative clinical features and microscopic analyses: **A -** Clinical aspect of an exophytic irregular yellowish and dark red-violaceous nodular lesion in right ventral and lateral border of the tongue; **B -** After 18 months of follow-up, no recurrence was noticed; **C -** Photomicrograph showing squamous epithelium with acanthosis, hyperparakeratosis, and elongated rete ridges, which encompassed dilated and congested vessels. (H&E, 10X); **D -** Photomicrograph presenting vascular spaces delimited by normal-appearing endothelial cells containing inside numerous erythrocytes and proteinaceous eosinophilic material. (H&E, 10X).

A 23-year-old man attended a private clinic complaining of an uncomfortable, slowly progressing mass on his tongue that impaired speech and swallowing and had been present for two weeks. During anamnesis, he mentioned that he used to smoke *Cannabis sativa*. He did not indicate comorbidities. He denied having a cough, runny nose, nasal congestion, or fever. The intraoral examination revealed a painless exophytic irregular dark red-violaceous nodular lesion in the tongue’s right ventral and lateral border and a similar papule in the ipsilateral dorsal region surrounded by white plaques (Figure 1A).

Under the clinical hypotheses of Kaposi’s sarcoma (KS) and COVID-19-related lesions, an incisional biopsy was performed. And a complete blood count and serology for Human Immunodeficiency Virus (HIV) 1 and 2, as well as a SARS-CoV-2 reverse-transcriptase-polymerase-chain-reaction test (RT-PCR test for COVID-19) were requested. Nasopharyngeal and oropharyngeal swabs were taken for RT-PCR test. None of the examinations showed abnormalities.

Microscopically, hyperparakeratosis, acanthosis, and papillomatosis in close association with large, dilated vessels lined by normal-appearing endothelial cells and containing inside erythrocytes and proteinaceous eosinophilic material were observed (Figure 1C-D).

**Figure 2:**
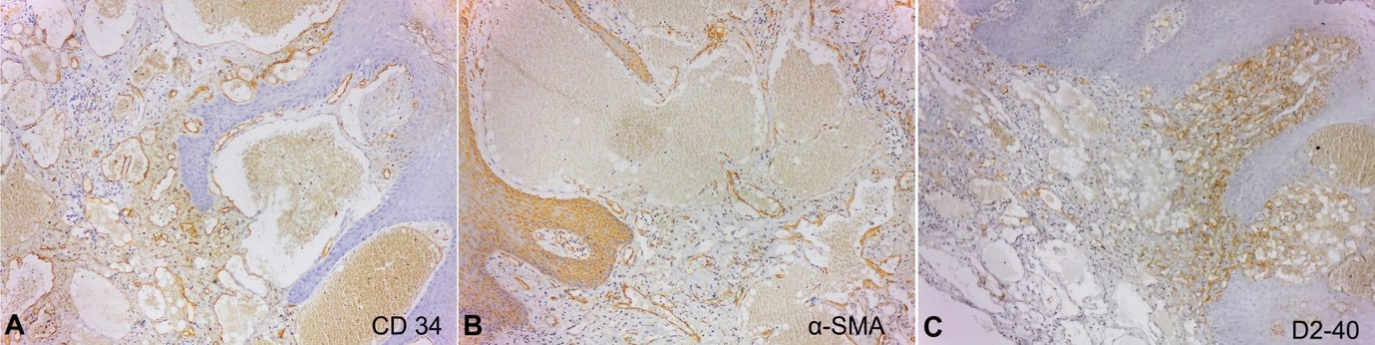
Immunohistochemical reactions. **A -** Most vessels showing positivity for CD34; **B** **-** Some vessels were highlighted by α-SMA; **C** **-** Focal positivity for D2-40. (DAB stain, 10X).

An immunohistochemical panel was performed. Most vessels were CD34 positive; some were highlighted by α-SMA, whereas D2-40 was focal (Figure 2A-C). Human Herpes Virus 8 (HHV-8) was negative.

Based on the clinical, microscopical, and immunohistochemical features, a definitive diagnosis of hemangiolymphangioma was obtained. The patient underwent minimally invasive surgical excision. After 18 months of follow-up, there were no signs of relapse (Figure 1B).

An electronic search was conducted in PubMed, Scopus, and Web of Science for studies published up to August 2022, with the following keywords: (“hemangiolymphangioma” OR “lymphangiohemangioma”) AND (“oral lesions” OR “mucosal lesions”). Related articles were also searched in the reference lists of the found full-text articles. 16 full-text articles were evaluated,^3,10,12,16-20^ of which two were excluded because the lesions were in the parotid gland and neck, respectively. Table 1 summarizes cases reported in scientific journals. Eight were located on the tongue,^5,6,8,9,16,19^ being 3 cases reported by Jian.^16^

**Table 1:** Published case reports^3-10,12,16-20^ on hemangiolymphangioma and lymphangiohemangioma of the oral cavity

Ref	Age (Y)	Sex	Site	Treatment Modality
^8^	7	M	Tongue	Transfixion
^17^	62	M	Mandible	Surgical enucleation with curettage
^16^	3-37	F/ M*	Tongue	Surgery
^5^	5	F	Tongue	Surgery
^9^	9	F	Tongue	Surgery
^7^	18	M	BM	Surgery
^18^	41	F	FoM	Surgery
^19^	6	M	FoM + Tongue	Surgery
^6^	2	M	Tongue	Surgery + Propranolol
^11^	26	M	BM	Surgery
^3^	21	F	BM	Surgery
^14^	46	F	Mandible	Surgery
^4^	5	M	BM	Surgery
^20^	5	F	BM	Surgery

* Data not stratified, 3 cases reported. BM= buccal mucosa; F= female; FoM= floor of the mouth; M= male;

Ref= reference; y=years.

## DISCUSSION

Hemangiolymphangioma occurring exclusively in the oral cavity is very scarce^1-3^ as the most frequent sites are the neck’s anterior and posterior cervical triangle.^2^ Moreover, a histological assessment may be challenging because of its similarity with other vascular lesions, such as lymphangioma, and angiokeratoma.^6,10,14^ Lymphangioma has been suggested to be the lymphatic counterpart of angiokeratoma,^10,15^ which demonstrates immunopositivity for CD31 and CD34.^10^ Hemangiolymphangiomas are mixed lesions, and like angiokeratomas, may present positive staining for some lymphatic markers, *i.e.*, CD31 and D2-40,^2^ which supports their lymphatic derivation. Consistent with previous reports,^2,10^ this specimen showed immunoreactivity for CD34 and focal positivity for D2-40.

In this case, it may be hypothesized that there were two etiopathogeneses. A vascular malformation may have developed from birth, although not apparent, and persisted until the patient noted the swelling.^3^ Conversely, the lesion could also be an acquired vascular malformation caused by mechanical trauma in an area with anomalous blood and lymphatic vessels.^14^ In hemangiolymphangiomas, the increased proliferative capacity of the stratified squamous epithelium seems to be a secondary reaction to vascular ectasia.^21^ Acanthosis, elongated rete pegs, and hyperparakeratosis encase dilated vessels, which may contain red blood cells, thrombi, and eosinophilic proteinaceous material.^1,5,8,11^

After the physical clinical examination, based on its features, such as an exophytic irregular dark red-violaceous nodular lesion, the clinical hypothesis of KS and a COVID-19-related lesion was made. The latter was because of the COVID-19 outbreak, in 2020. Some authors published the COVID-19-related vascular alteration of the tongue,^13^ similar to our report. KS is the most common neoplasm associated with HIV. In nearly 20% of patients, the initial manifestation of HIV infection is in the mouth. Therefore, we ruled out all these clinical hypotheses.

In a multicenter study of oral lymphatic malformations, disagreement between clinical and histopathological diagnoses was encountered in 58.2% of the lesions.^22^ Considering that vascular lesions within the mouth have a broad etiopathogenesis, the final diagnosis of such entities may be challenging.^23^ To improve the diagnostic process and establish a definite diagnosis of an uncommon lesion, this study described the immunohistochemical profile of an oral hemangiolymphangioma.

A thorough evaluation of a vascular lesion was presented and the diagnosis of hemangiolymphangioma of the tongue was assessed by histopathological and immunohistochemical analysis. Well-documented cases of hemangiolymphangioma are important for further categorization of mixed vascular malformations.
